# Establishment of age-stratified serum vitamin B6 pyridoxal and pyridoxic acid reference intervals among children aged 1–16 years in Henan, china

**DOI:** 10.3389/fnut.2025.1592045

**Published:** 2025-07-30

**Authors:** Yusheng Luan, Nan Chen, Xucheng Wang, Xiaojuan Li, Junmei Yang, Tiewei Li

**Affiliations:** Department of Clinical Laboratory, Zhengzhou Key Laboratory of Children’s Infection and Immunity, Henan Children’s Hospital, Children’s Hospital Affiliated to Zhengzhou University, Zhengzhou, Henan, China

**Keywords:** vitamin B6 pyridoxal, vitamin B6 pyridoxic acid, reference intervals, children, Henan

## Abstract

**Background/Objective:**

The development of population-specific reference intervals (RIs) for serum vitamin B6 (B6) in Chinese pediatric populations has not been established. Current clinical practice relies on international thresholds, but diagnostic sensitivity with domestic biochemical profiles remains suboptimal. This study aims to establish age-stratified reference intervals for serum vitamin B6 pyridoxal (B6-PL) and pyridoxic acid (B6-PA) in Henan children aged 1–16 years.

**Methods:**

This retrospective study analyzed 1,458 healthy children undergoing routine physical examinations at Henan Children’s Hospital from December 2021 to June 2024. Serum B6-PL and B6-PA were quantified using the Xevo TQ-S Micro liquid chromatography-tandem mass spectrometry system (Waters Corporation). Non-normally distributed data are expressed as median interquartile range (IQR) [M (P_25_, P_75_)]. Age- and sex-related differences were assessed using Mann-Whitney U or Kruskal-Wallis tests.

**Results:**

Analysis revealed that age significantly influences serum levels of B6-PL and B6-PA in children, while gender showed no significant effect. The pediatric cohort demonstrated a male predominance (58.98%, 860/1458) with a notable prepubertal skew (93.28%, 1360/1458 aged < 12 years). Further analysis facilitated the establishment of RIs for B6-PL and B6-PA across different age groups. The RIs for B6-PL were 1.62–17.70 ng/mL for children aged 1–5 years, 1.39–15.15 ng/mL for children aged 6–11 years, and 1.06–14.30 ng/mL for children aged 12–16 years. The RIs for B6-PA were 1.20–5.46 ng/mL for children aged 1–5 years, 1.10–4.41 ng/mL for children aged 6–11 years, and 0.95–4.99 ng/mL for children aged 12–16 years.

**Conclusion:**

This study establishes the first age-stratified RIs for serum vitamin B6 metabolites (B6-PL and B6-PA) among children aged 1–16 years in Henan, China.

## 1 Introduction

Vitamin B6 serves as a critical cofactor in pediatric neurodevelopment and systemic growth, supporting neurotransmitter biosynthesis (e.g., serotonin, GABA), amino acid metabolism, and myelination. Defining precise RIs for vitamin B6 is essential to establish clinically actionable thresholds for identifying deficiency or toxicity. Pediatric B6 deficiency is associated with pathologies including B6-dependent epilepsy (1), hyperhomocysteinemia (HHC) (2, 3), and systemic inflammatory responses (4), while prolonged elevation may manifest as dermatological rashes, gastrointestinal disturbances (e.g., diarrhea, vomiting), or neurological sequelae. These dual risks underscore the necessity of establishing validated pediatric RIs to guide evidence-based clinical decision-making and mitigate adverse outcomes.

Vitamin B6, also known as pyridoxine, is a light-sensitive and thermolabile water-soluble vitamin that serves as a critical coenzyme in human physiology. It comprises six interconvertible pyridine derivatives: pyridoxine (PN), pyridoxamine (PM), pyridoxal (PL), and their phosphorylated forms (PNP, PMP, and PLP) (5–9). The oxidation product, pyridoxic acid (PA), serves as a biomarker for assessing short-term B6 intake (10). While liquid chromatography-tandem mass spectrometry (LC-MS/MS) remains the gold standard for quantifying B6 metabolites due to its rapidity and precision, clinically significant discrepancies persist between manufacturer-provided RIs and empirical pediatric data. This underscores the need for population-specific RIs tailored to Chinese children.

The development of pediatric vitamin B6 RIs present significant methodological challenges due to age-dependent physiological variations, sex-specific metabolic profiles, and modifiable confounders such as dietary patterns and pharmacological exposures. To address these complexities, we performed a retrospective analysis of biochemical datasets to quantify covariate effects on B6 homeostasis, enabling the derivation of age-stratified RIs for Henan children. This covariate-adjusted approach provides critical evidence for establishing national pediatric B6 standards while offering a reproducible framework for region-specific RI determination across diverse populations in China.

## 2 Materials and methods

### 2.1 Study design and participants

This was a retrospective study that analyzed deidentified clinical data from children undergoing routine health assessments at Henan Children’s Hospital (Children’s Hospital Affiliated to Zhengzhou University) between December 2021 and June 2024. Participants were included if they were aged 1–16 years and had complete records of routine health examinations. Exclusion criteria included: (1) presence of malignant tumors or hematologic disorders, (2) major congenital anomalies or active infections, (3) metabolic conditions affecting vitamin B6 homeostasis, (4) acute neurological disorders such as encephalitis, febrile seizures, or status epilepticus, and (5) gross hemolysis observed in serum samples. The study protocol, approved by the Ethics Review Committee of Henan Children’s Hospital (Approval NO. 2022-K-L045) complied with the Declaration of Helsinki. As the analysis utilized anonymized retrospective data obtained during standard clinical care, informed consent requirements were exempted, as confirmed by the Hospital Ethics Review Board of Henan Children’s Hospital (Approval NO. 2022-K-L045).

### 2.2 Clinical data collection

Demographic and biochemical data, including age, sex, and serum vitamin B6 pyridoxal (B6-PL) and vitamin B6 pyridoxic acid (B6-PA) concentrations, were retrospectively obtained from institutional electronic medical records (EMRs). B6-PL and B6-PA levels were measured using a Waters ACQUITY UPLC I-Class Plus/Xevo TQ-S Micro tandem mass spectrometry system (Waters Corporation). Serum samples were processed following the manufacturer’s specifications (Hanahao Biotech, Tianjin, China), employing solid-phase extraction followed by chromatographic separation of 5 μL aliquots.

Mass spectrometric analysis utilized electrospray ionization in positive ion mode (ESI+) with the following operational parameters: capillary voltage set at 3.0 kV, ion source temperature at 150°C, desolvation temperature at 500°C, desolvation gas flow at 1000 L/h, and cone gas flow at 50 L/h. Quantitative analysis was performed using multiple reaction monitoring (MRM) facilitated by MassLynx v4.2 software, with analyte-specific transitions calibrated against certified reference materials.

Quantitative limits were established at 0.5 ng/mL for B6-PA and 0.6 ng/mL for B6-PL, with precision and accuracy verified across 20 independently prepared replicates. Maximum concentration deviation was 11.10%, and the coefficient of variation (CV) did not exceed 4.25%, conforming to the acceptance criteria of ± 15% deviation and CV ≤ 20%. Intra- and inter-day precision were assessed at three concentration levels (B6-PA: 1.5, 10, 75 ng/mL; B6-PL: 1.8, 12, 90 ng/mL) over three consecutive days, with one batch analyzed per day. Intra-day and inter-day CVs were ≤ 7.10% and 8.61%, respectively, satisfying the threshold of ≤ 15%. Recovery evaluation was performed using baseline clinical samples spiked at the aforementioned concentrations, yielding recovery rates between 0.854 and 1.11, meeting the acceptable range of 0.85–1.15.

### 2.3 Statistical analysis

Statistical analyses were conducted using SPSS version 27.0. The Kolmogorov-Smirnov test evaluated the normality of continuous variable distributions. Non-normally distributed data are expressed as median (P_25_, P_75_). Descriptive statistics were employed to depict the distribution of B6-PL and B6-PA across various age groups, sexes, and seasonal testing periods. The Mann-Whitney U test was utilized for pairwise comparisons, while the Kruskal-Wallis test assessed differences among multiple groups. Data visualization was performed using GraphPad Prism 8 (GraphPad Software Inc.). A two-sided *P*-value of less than 0.05 was deemed statistically significant.

## 3 Result

### 3.1 Participants characteristic

The levels of B6-PL and B6-PA were summarized using descriptive statistics (Table 1). B6-PL and B6-PA serve as distinct biomarkers derived from the same child’s sample. Among the 1,458 participants, boys comprised a larger proportion, representing 58.98% of the overall population. As indicated in Table 1, the majority of children were under the age of 12, with those aged < 12 years constituting 93.27% of the total, while children aged ≥ 12 years accounted for only 6.7%. Additionally, the minimum and maximum concentrations of B6-PL were 0.64 ng/mL and 19.98 ng/mL, respectively. For B6-PA, the corresponding values ranged from 0.34 ng/mL to 6.00 ng/mL.

**TABLE 1:** Characteristics of B6-PL and B6-PA.

Variables	*n* (%)	B6-PL (ng/mL)		B6-PA (ng/mL)	
		Min	Max	Min	Max
Gender					
Boy	860 (58.98%)	0.73	19.98	0.66	5.96
Girl	598 (41.02%)	0.64	19.78	0.34	6.00
Age (years)					
1–5	669 (45.88%)	1.17	19.98	0.34	6.00
6–11	691 (47.39%)	0.64	18.72	0.67	5.74
12–16	98 (6.7%)	0.80	16.00	0.79	5.44
Total	1,458 (100%)	0.64	19.98	0.34	6.00

B6-PL, vitamin B6 pyridoxal; B6-PA, vitamin B6 pyridoxic acid.

### 3.2 Gender variation in B6-PL and B6-PA levels

**FIGURE 1:**
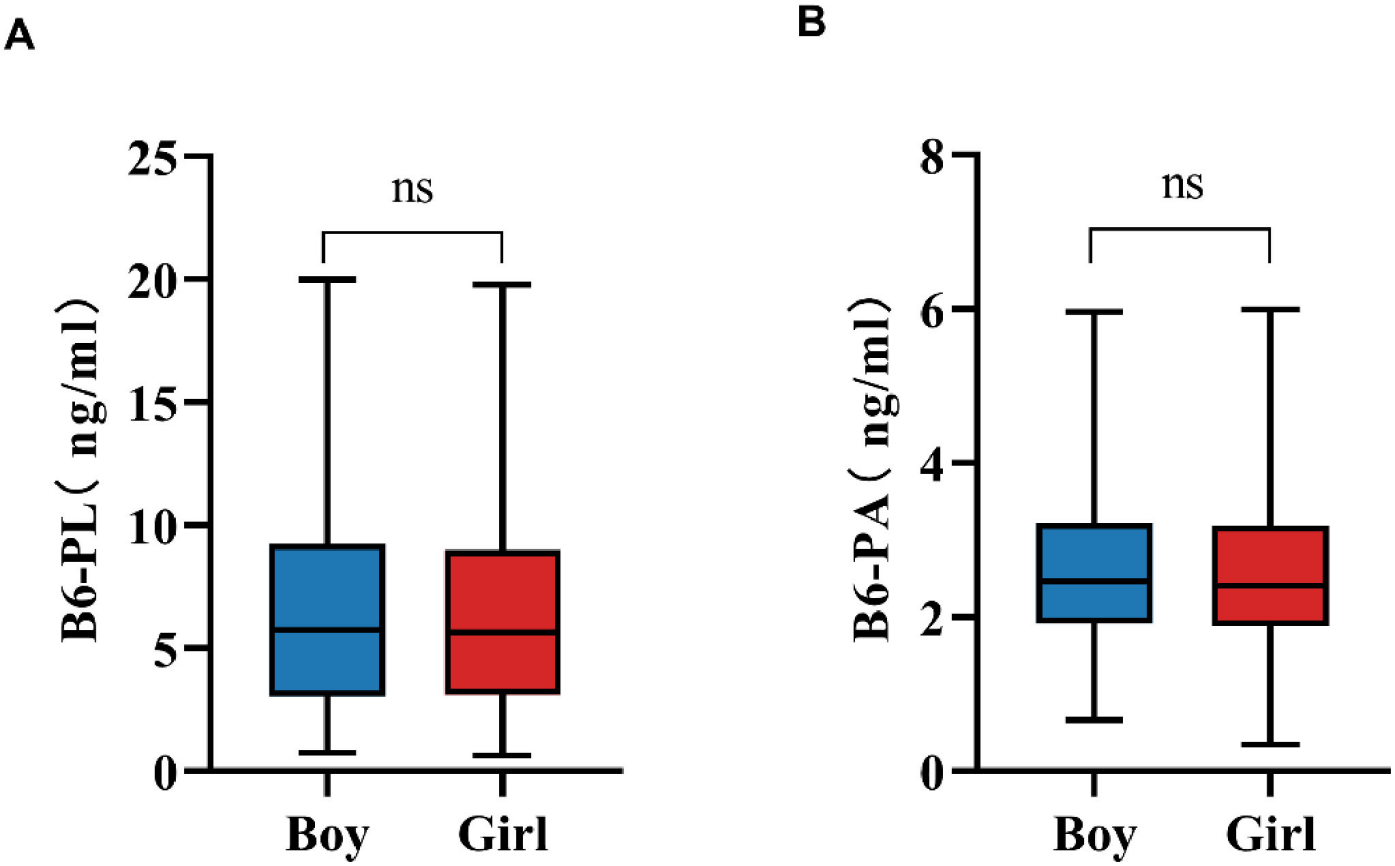
Gender variation in B6-PL **(A)** and B6-PA **(B)** level. * *P* < 0.05, ** *P* < 0.01, *** *P* < 0.001; ns, no significant.

Gender-specific differences in B6-PL and B6-PA levels are summarized in Table 2, which details the distributions of these vitamin B6 biomarkers. Median concentrations were comparable between sexes: boys exhibited B6-PL levels of 5.73 ng/mL (IQR: 3.03–9.24), while girls had 5.63 ng/mL (IQR: 3.12–8.98). Similarly, B6-PA concentrations showed no clinically significant divergence between sexes, with medians of 2.46 ng/mL (IQR: 1.92–3.22) in boys and 2.41 ng/mL (IQR: 1.88–3.19) in girls. As shown in Figure 1, statistical analysis revealed no significant differences in biomarker levels between sexes (*P* > 0.05).

**TABLE 2:** Gender variation in B6-PL and B6-PA level.

Variables	Boy (*n* = 860)			Girl (*n* = 598)			*Z*	*P*
	P_25_	P_50_	P_75_	P_25_	P_50_	P_75_		
B6-PL (ng/mL)	3.03	5.73	9.24	3.12	5.63	8.98	0.180	0.857
B6-PA (ng/mL)	1.92	2.46	3.22	1.88	2.41	3.19	−0.655	0.512

B6-PL, vitamin B6 pyridoxal; B6-PA, vitamin B6 pyridoxic acid.

### 3.3 Age variation in B6-PL and B6-PA levels

**FIGURE 2:**
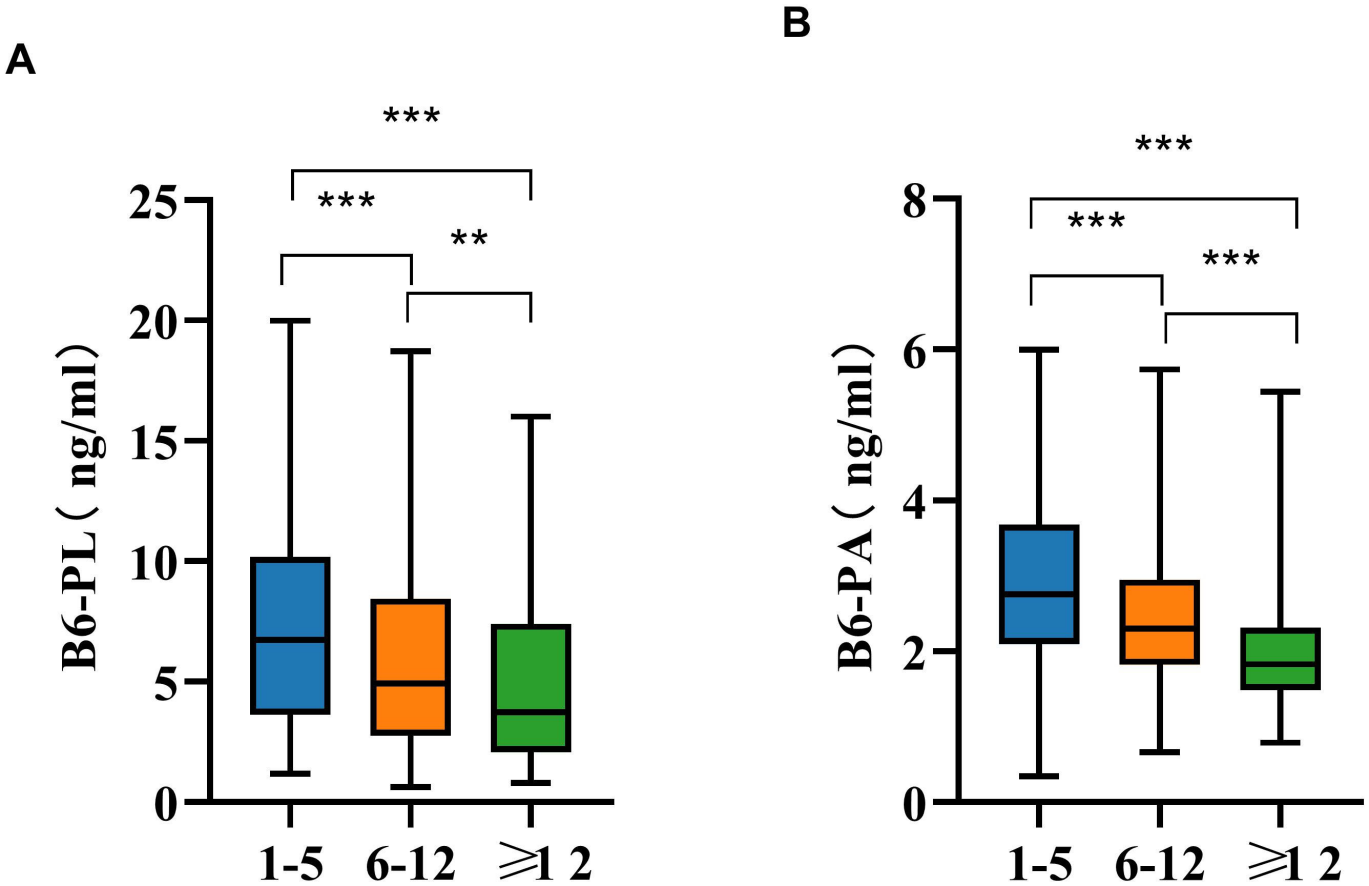
Age variations in B6-PL **(A)** and B6-PA **(B)** in pairwise comparisons. * *P* < 0.05, ** *P* < 0.01, *** *P* < 0.001; ns, no significant.

Age-related differences in B6-PL and B6-PA concentrations are detailed in Table 3. Participants were stratified into three age categories: 1–5 years, 6–11 years, and ≥ 12 years. Both biomarkers exhibited significant variation across these groups (*P* < 0.001), with the youngest cohort (1–5 years) demonstrating the highest median levels of B6-PL [6.74 ng/mL (IQR: 3.64–10.15)] and B6-PA [2.75 ng/mL (IQR: 2.10–3.68)]. Concentrations of both biomarkers decreased progressively with age (*P* < 0.001; Figure 2). Pairwise comparisons confirmed this trend: B6-PL declined from 6.74 ng/mL (1–5 years) to 4.93 ng/mL (6–11 years; *P* < 0.001) and further to 3.74 ng/mL (≥ 12 years; *P* < 0.05 vs. 6–11 years; *P* < 0.001 vs. 1–5 years). Similarly, B6-PA decreased from 2.75 ng/mL (1–5 years) to 2.30 ng/mL (6–11 years; *P* < 0.001) and stabilized at 1.83 ng/mL (≥ 12 years; *P* < 0.001 vs. younger groups).

**TABLE 3:** Age variations in B6-PL and B6-PA levels.

Variables	1–5 (*n* = 669)			6–11 (*n* = 691)			≥ 12 (*n* = 98)			*Z*	*P*
	P_25_	P_50_	P_75_	P_25_	P_50_	P_75_	P_25_	P_50_	P_75_		
B6-PL (ng/mL)	3.64	6.74	10.15	2.77	4.93	8.44	2.10	3.74	7.36	59.446	<0.001
B6-PL (ng/mL)	2.10	2.75	3.68	1.83	2.30	2.95	1.50	1.83	2.30	121.453	<0.001

B6-PL, vitamin B6 pyridoxal; B6-PA, vitamin B6 pyridoxic acid.

### 3.4 Reference intervals of B6-PL and B6-PA according to age and gender group

Table 4 presents age-stratified RIs for B6-PL and B6-PA in a non-sex-stratified pediatric cohort, calculated using non-parametric percentile methods (2.5th–97.5th). For B6-PL, RIs declined with age: 1.62–17.70 ng/mL (1–5 years), 1.39–15.15 ng/mL (6–11 years), and 1.06–14.30 ng/mL (≥ 12 years). Similarly, B6-PA RIs were 1.20–5.46 ng/mL (1–5 years), 1.10–4.41 ng/mL (6–11 years), and 0.95–4.99 ng/mL (≥ 12 years).

**TABLE 4:** Recommended reference intervals for different age groups (P_2.5_, P_97.5_)*.

Variables	1–5	6–11	≥12
B6-PL (ng/mL)	1.62–17.70	1.39–15.15	1.06–14.30
B6-PA (ng/mL)	1.20–5.46	1.10–4.41	0.95–4.99

*No significant sex-based differences. B6-PL, vitamin B6 pyridoxal; B6-PA, vitamin B6 pyridoxic acid.

## 4 Discussion

Vitamin B6, a water-soluble micronutrient essential for cellular function, exists primarily as pyridoxal 5′-phosphate (PLP), its bioactive coenzyme form (11). Bioinformatic analyses reveal that PLP serves as an essential cofactor for over 4% of human enzymes, with significant statistical enrichment observed in amino acid-metabolizing enzymes–particularly transaminases and decarboxylases–highlighting its central role in nitrogen metabolism (12–14). As mammals, including humans, cannot synthesize vitamin B6 endogenously, dietary intake remains the primary source, with minimal contributions from gut microbiota synthesis (12, 15, 16). Given its critical role in metabolic homeostasis, establishing reliable RIs for vitamin B6 biomarkers is clinically imperative. PL serves as the exclusive direct precursor for PLP biosynthesis, making its adequate availability essential for PLP production. Abnormal PL levels may indicate PLP deficiency or enzymatic dysfunction. Serum PL reflects dietary vitamin B6 intake and is thus suitable for assessing nutritional status, particularly in children with diverse dietary patterns (e.g., infants and preschoolers) where subclinical B6 deficiency may occur (17). Conversely, PA represents the terminal catabolite of vitamin B6 metabolism and functions as a biomarker of metabolic turnover.

This study found no significant sex-based differences in B6-PL or B6-PA levels across pediatric age groups, suggesting shared RIs for boys and girls. This outcome may reflect a region-specific phenomenon rather than a nationwide pattern, yet it provides a valuable reference point for other regions and highlights the importance of localized evaluation in nutritional biomarker research. The observed uniformity may reflect heightened demand for vitamin B6 during growth spurts, driving balanced nutritional intake among children irrespective of sex.

Age-stratified analysis revealed inverse correlations between vitamin B6 levels and age. The 1–5 years cohort exhibited the highest median B6-PL and B6-PA concentrations, with progressive declines in older groups. This finding underscores the necessity for stage-specific monitoring and intervention of vitamin B6 levels throughout the life course. This is particularly critical during childhood, a period marked by rapid growth and physiological development, during which B6 concentrations exhibit considerable variability. This trend aligns with vitamin B6’s role in neurodevelopment, where it acts as a coenzyme for neurotransmitter synthesis and supports rapid neural maturation in early childhood (18–20). In addition, children’s basal metabolic rate gradually increases with age. PLP is an essential coenzyme for the conversion of glycogen to glucose-1-phosphate, so serum B6 levels tend to decrease as age increases. Regarding lipid metabolism, vitamin B6 is involved in the synthesis of arachidonic acid, a key substance for neural development. During growth, children have a high demand for protein, which requires PLP as a coenzyme to promote protein synthesis and support growth and development. In summary, the accelerated metabolism of proteins, lipids, and carbohydrates during growth increases the utilization of vitamin B6, leading to a decline in serum B6 levels with age (21). Furthermore, parents often prioritize nutrient-dense diets for younger children, while older individuals may develop selective eating habits, resulting in reduced B6 intake and subsequently lower serum B6 levels.

Based on the influence of age and gender, this study established RIs for B6-PL and B6-PA in Henan children. These RIs improve diagnostic precision for B6 deficiency or excess, mitigating risks of misdiagnosing conditions like anemia, immunocompromise, or delayed surgical recovery linked to B6 dysregulation.

However, this study has certain limitations. One notable shortcoming in the establishment of our RIs is the lack of a comprehensive analysis of B6 forms. Specifically, PLP, the biologically active coenzyme form of B6, was not included in our current detection panel. We acknowledge this limitation and plan to incorporate PLP measurements in future work to further refine and improve the accuracy of our RIs. During the establishment of the RIs, individual differences were not fully considered. For instance, factors such as children’s dietary habits and picky eating behaviors may affect serum B6 concentrations. Additionally, this study did not further classify RIs by season, as the existing sample size was insufficient to support such an analysis. Future research should aim to verify these influencing factors with a larger sample size. Moreover, this study only covers Henan Province, and to expand the findings to a national level, more sample data from other regions will be necessary to refine the RIs.

## 5 Conclusion

Age-stratified variations in vitamin B6 status were systematically characterized through LC-MS/MS quantification, establishing region-specific reference intervals for pediatric populations in Henan, China. The cohort was stratified into three developmental phases: 1–5 years, 6–11 years and ≥ 12 years, with corresponding RIs for B6-PL (1.62–17.70, 1.39–15.15, 1.06–14.30 ng/mL) and B6-PA (1.20–5.46, 1.10–4.41, 0.95–4.99 ng/mL). These age-partitioned thresholds provide reference for addressing region-specific nutritional requirements and enable evidence-based clinical decision-making in pediatric metabolic disorder prevention.

## Data Availability

The original contributions presented in this study are included in this article/supplementary material, further inquiries can be directed to the corresponding authors.
